# A Cloud-Based Car Parking Middleware for IoT-Based Smart Cities: Design and Implementation

**DOI:** 10.3390/s141222372

**Published:** 2014-11-25

**Authors:** Zhanlin Ji, Ivan Ganchev, Máirtín O'Droma, Li Zhao, Xueji Zhang

**Affiliations:** 1 Telecommunications Research Centre, University of Limerick, Limerick, Ireland; E-Mails: Zhanlin.Ji@ul.ie (Z.J.); Mairtin.ODroma@ul.ie (M.O.); 2 Research Institute of Information Technology, Tsinghua University, Beijing 100080, China; E-Mail: Zhaoli@tsinghua.edu.cn; 3 Research Center for Bioengineering and Sensing Technology, University of Science and Technology Beijing, Beijing 100080, China; E-Mail: Zhangxueji@ustb.edu.cn

**Keywords:** Internet of Things (IoT), smart cities, Always Best Connected and best Served (ABC&S), Intelligent Transport Systems (ITS), car parking, middleware, Hadoop

## Abstract

This paper presents the generic concept of using cloud-based intelligent car parking services in smart cities as an important application of the Internet of Things (IoT) paradigm. This type of services will become an integral part of a generic IoT operational platform for smart cities due to its pure business-oriented features. A high-level view of the proposed middleware is outlined and the corresponding operational platform is illustrated. To demonstrate the provision of car parking services, based on the proposed middleware, a cloud-based intelligent car parking system for use within a university campus is described along with details of its design, implementation, and operation. A number of software solutions, including Kafka/Storm/Hbase clusters, OSGi web applications with distributed NoSQL, a rule engine, and mobile applications, are proposed to provide ‘best’ car parking service experience to mobile users, following the Always Best Connected and best Served (ABC&S) paradigm.

## 1. Introduction

**Figure 1.**
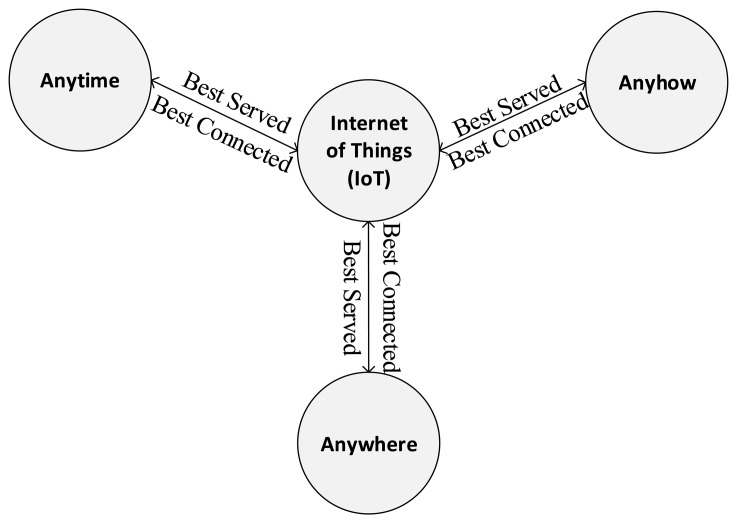
A high-level view of the Internet of Things.

In September 2009, the European Union (EU) endorsed an Internet of Things (IoT) Strategic Research Roadmap, proposed by the Cluster of European Research Projects (CERP), named CERP-IoT [1], with the purpose of promoting, sharing and propagandizing the research projects and related research outcomes in the IoT area, especially the application of sensor technology in IoT, such as Intelligent Transport Systems (ITS) [2], family-domain smart eHealth/mHealth, wearable sensing and computing, green buildings, smart homes, smart cities, *etc*. The report formally proposes a new communication dimension of information and communication technology (ICT)—Maintained at any time, in anyplace, by anything and anyone, providing any service within any network. In fact, this is an extension and further development (bringing it to a new level) of the “anytime-anywhere-anyhow” communication paradigm [3,4], Figure 1. In the public tender announcement (for a 7 billion euro annual financial support) of the EU Seventh Framework Programme (FP7), multiple projects related to IoT in nearly 20 areas were supported.

**Figure 2.**
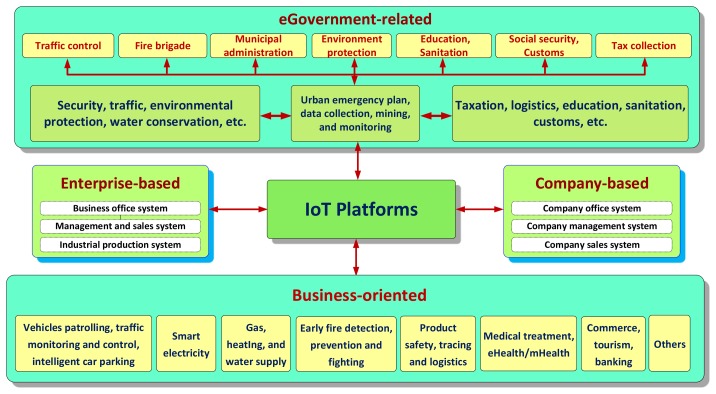
The IoT platform types.

The IoT platforms could be categorized into four types: eGovernment-related, production/ enterprise-based, company-based, and pure business-oriented platforms. Figure 2 illustrates these four types of the IoT platforms, which are described in more detail below.

The eGovernment IoT is the supporting foundation that should facilitate the city's/region's/state's economic development and management. It is usually funded through a government's public welfare scheme, which gradually promotes the development of eGovernment towards the IoT. The eGovernment IoT platform needs information relevance to prevent the ‘information island’ effect and to ensure network- and information security.

The enterprise-based and company-based IoT platforms can improve competitiveness and service assurance of market-oriented enterprises and companies. These require independently funded IoT projects for more efficient production management, warehousing, distribution, transportation, logistics, marketing, and supply chain management.

The pure business-oriented IoT platforms may become an important facilitator as optimization and integration of information market resources require involving all end-users, network providers, and service providers to build a modern industry established on valuable IoT business models and platforms.

The CERP-IoT report [1] predicted that the automotive industry will be using ‘smart things’ to monitor everything. Especially when using wireless technology for vehicle-to-infrastructure (V2I) communication, the real-time locating systems (RTLS) can enable tracking and tracing services, which will significantly advance the ITS applications. The intelligent car parking systems, such as the one described in this paper, constitute an important part of the ITS with a primary purpose to find, allocate, reserve, and provide the ‘best’ available car parking lot to each individual user who is driving a car in a particular area. Valuable add-on functionality could be the provision of navigation instructions to the driver for reaching the lot. These systems may serve as a foundation and a common business model for a generic IoT operational platform due to their properties of providing pure business-oriented services.

Researchers in [5] show that more than 66% of drivers do not mind paying for car parking facilities during their working hours. This directly adds value to the car parking business, which is a stimulus for the development of intelligent car parking services for smart cities.

The existing car parking systems are not very efficient as they do not provide the ‘best’ service, e.g., finding the nearest available car parking lot. At the sensor layer, most of researchers today focus on detecting the car-parking-lot occupancy. In [6], a car-parking-lot detection method is proposed based on an automatic threshold algorithm; as the image processing algorithms are expensive, a hardware solution is suggested. In [7], sensors are used for intelligent autonomous parking. In [8], laser scanners are used to retrieve the car-parking-lot position.

At the communication layer, an InfoStation-based multi-agent system facilitating a car parking locator service is proposed in [9]; users are provided with a personalized service based on their location and mobile device's capabilities. In [10], a wireless sensor network solution is proposed for car parking management along with a routing protocol for improving the transport reliability.

At the application layer, research efforts are usually focused on one particular aspect. For instance, with respect to car routing, a route planning for ITS is proposed in [11] for reducing the number of accidents involving vehicles with dangerous cargo. As regards reducing the driver's waiting time, a corresponding access control system is proposed in [12]. With regard to the driver's behavior, an agent-based behavior algorithm is proposed in [13] for seeking the optimal car parking lot. As regards the cloud aspect, a cloud-based computing model for ITS is proposed in [14]. All these examples, however, lack an end-to-end solution for intelligent car parking services in the ‘big data’ age.

As the low-powered processing chips, smart mobile devices, cloud computing, future networks (NGN) [15] and communication environments, such as the Ubiquitous Consumer Wireless World (UCWW) [3,4] develop rapidly, there is a significant opportunity for the development of intelligent car parking systems, which can serve the users in an Always Best Connected and best Served (ABC&S) manner [16]. This paper describes such a system, which is established on a cloud-based infrastructure and follows the ‘anytime-anywhere-anyhow’ communication paradigm [3,4].

## 2. IoT Intelligent Car Parking System

**Figure 3.**
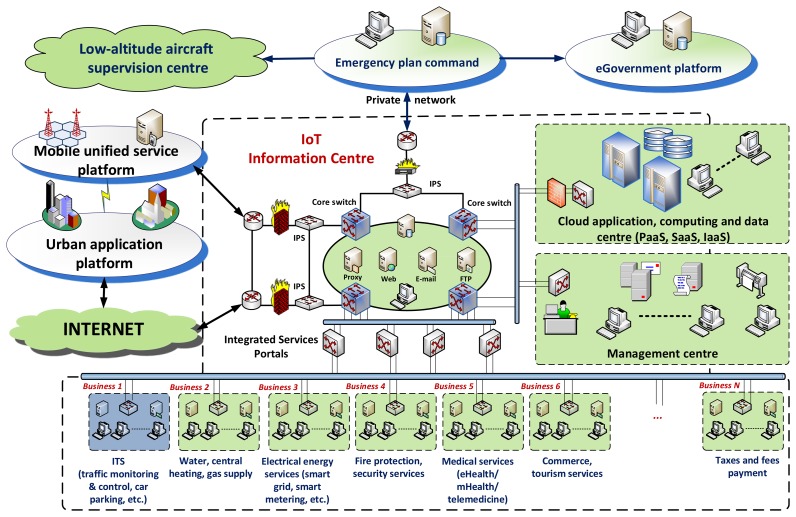
A high-level view of an IoT-based Smart City.

ITS and other systems, such as electrical energy systems, water-, heating- and gas supply systems, city fire protection and security systems, eHealth/mHealth systems, *etc.*, provide intelligent IoT services to make the city smarter [17]. ITS locates in the business layer of IoT, communicates with the cloud-based information centre of the smart city, and delivers ‘best’ transport-related services to users, such as traffic monitoring & control, route planning, car parking service, *etc*. Figure 3 depicts a high-level view of a centralized IoT platform, which could serve as a generic architectural foundation for a ‘smart city’ establishment, operation, administration, and management. This top-level generic architectural design can unify the development of business applications as an efficient and economical process. For instance, if an ITS service provider wants to deploy a car parking service within a smart city's shared cloud infrastructure, it will only need to focus on the operating model to realize the smartness of the car parking.

**Figure 4.**
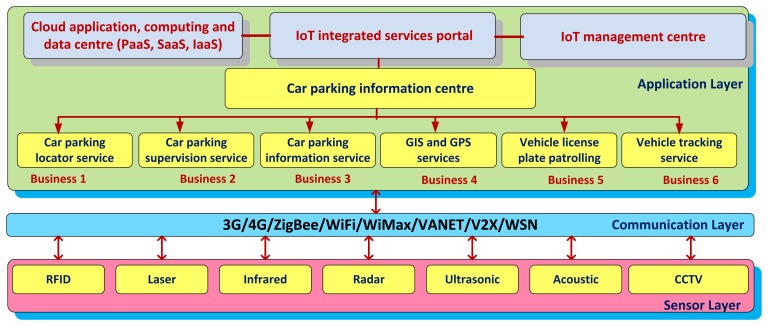
An IoT intelligent car parking system for a Smart City.

Here we propose an intelligent car parking system for integration into a smart-city IoT architecture, which consists of three layers—A sensor layer, a communication layer, and an application layer (Figure 4).

At the application layer, an information centre provides cloud-based services [18], *i.e.*, Platform as a service (PaaS), Software as a service (SaaS), and Infrastructure as a service (IaaS); *i.e.*, for allocating computing/storage resources for different car parking services. An IoT management centre administrates the smart city via an IoT integrated services portal. At the bottom, a number of business services explore a common interface to the communication layer. These include car parking locator, supervision, and information services, GIS/GPS services, vehicle license plate patrolling, car tracking services, *etc*.

At the communication layer, various wireless technologies provide connection between the application- and the sensor layer, based on the ABC&S communication paradigm. A 3-tier InfoStation-based network architecture [10] could be integrated in this layer to enable ‘anytime-anywhere-anyhow’ communication functionality in smart cities.

Different sensing technologies could be utilized at the sensor layer for embedded parking solutions, such as the Radio Frequency Identification (RFID) for car parking access control; laser, passive infrared, microwave radar, ultrasonic, passive acoustic array sensors, or Closed-Circuit Television (CCTV) with video image processing for detecting the status of the car parking lots; license plates with installed 3G/4G communication module for cars' tracking and tracing; *etc*.

**Figure 5.**
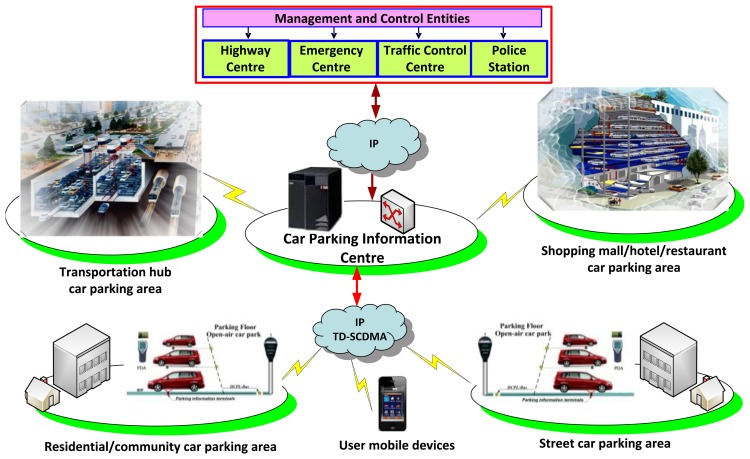
The intelligent car parking services' operational platform.

To enable the car parking system to work as an operational platform in a smart city, different car parking areas must be distinguished in providing ‘best’ car parking lots by executing different business roles and applications. Based on their properties, the car parking areas could be divided into four main categories (Figure 5): A transportation hub area; a residential/community area; a ground/street area; and a shopping mall/hotel/restaurant area. The relevant management and control entities, including a highway centre, emergency centre, traffic control centre, and police can get access to the information managed by the car parking information centre with high authority. The sensors deployed in the car parking area periodically send updated information as regards occupancy of the car parking lots to the car parking meters, which push this data to the information centre.

Users can interact with the system by installing the corresponding car parking application on their mobile devices. Facilitated by a personal assistant agent, each user can set up a personal profile which will be used by the application for finding, allocating, reserving, and paying for the ‘best’ parking lot in each particular scenario. Stored in the memory-based No-SQL database [19], the user profile will be dynamically updated to reflect changes in the user's context and behavior, which are analyzed by the system. With efficient car parking lot allocation algorithms/rules, the system is always able to provide the mobile user with the ‘best’ available car parking lot following the ABC&S paradigm.

## 3. Sample Car Parking System for University Campus

Every big university has a number of different car parking areas, e.g., for visitors, students, staff members, *etc*. University car parking belongs to the category of a residential/community car parking. Every working day, students and staff members spend usually a lot of time just to find an available car parking lot. This is not only a time-consuming and energy-wasteful process but it may also cause car traffic jams. With the intelligent cloud-based car parking service proposed here, an efficient utilization of available car parking facilities could be achieved within a ‘smart university’ environment.

**Figure 6.**
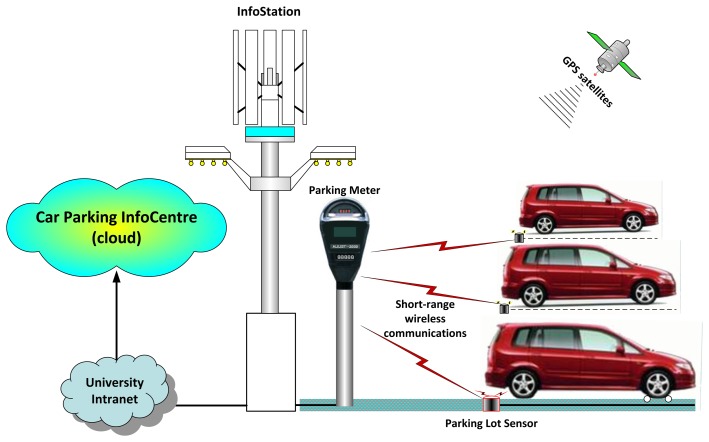
An infoStation-based university car parking system.

One way to achieve this is to have each car parking lot equipped with a sensor which is able to sense the presence of a car in it. An information station (InfoStation), operating in the car parking area, periodically collects and aggregates the car presence information from all sensors deployed in the area, e.g., by means of Wi-Fi, ZigBee, or other short-range wireless technology. In the case of paid car parks, optional parking meters could operate between the InfoStation and the sensors. When the occupation status of a car parking lot is changed, information about this is pushed by the InfoStation to the car parking Information Centre (InfoCentre) in the cloud via the university Intranet (Figure 6). Further in this paper, we are focusing only on the software implementation of this system.

### 3.1. Design

**Figure 7.**
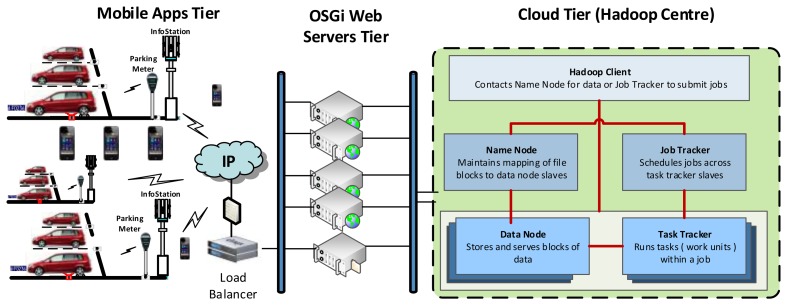
A high-level view of a cloud-based car parking system's application layer.

From the high-level view of the IoT-based smart city and the layered intelligent car parking system (Figures 3 and 4), the car parking service's application layer, e.g., within a university campus, could be deployed with three tiers, as shown in Figure 7. In the cloud tier, web applications serialize data into the Hadoop [20] centre; in the web servers tier, applications—developed as *Bundles*—dynamically register themselves on the Open Service Gateway initiative (OSGi) framework [21]; and in the mobile apps tier, mobile devices access the web applications and provide ‘best’ car parking services to their users.

#### 3.1.1. Cloud Tier

The cloud provides data storage and computing resources for the car parking service. It stores the ‘big data’ of available car parking lots, car parking area, cars' location, users' location and profiles, *etc*. The most recent data is usually stored in the Hadoop's Hbase [22] database to support real-time queries, whereas the history data is serialized to Hive [23] (a warehousing in Hadoop). For computing, a number of Map/Reduce algorithms [24] are used, such as a recommendation algorithm for suggesting the ‘best’ car parking lots to users, a recommendation algorithm based on friends' car parking suggestions, a profile-updating algorithm based on users' parking history, *etc*. To build an efficient and scalable system, a rule engine Drools [25] is used to make decisions, based on facts, quickly and reliably.

#### 3.1.2. OSGi Web Servers Tier

This tier acts as a bridge between the mobile apps tier and the cloud tier. Considering the great number of web applications/services running in this tier, the deployment of a new/updated application should be possible without stopping/restarting the corresponding web container/server. The OSGi provides an environment to modularize web applications into bundles, which can dynamically register themselves in the bundle's execution context. To provide high-performance and on-demand car parking services for users, a key-value based NoSQL database—Redis [26]—is used in this tier to provide scalable and distributed job queues. To optimize web resources' utilization, a load balancer distributes the user's requests across the cluster of web servers. A distributed system collects the web servers' log data and sends it to the cloud.

#### 3.1.3. Mobile Applications Tier

The Allied Business Intelligence (ABI) Research [27] reported that the Android operating system [28] played a dominant role in the smartphone market in 2013 (with 81% share). Thus the first version of the car parking mobile application is developed for Android mobile devices. When a user approaches the University campus, an automatic request is sent by the application (on behalf of the user) to an OSGi car parking web server asking for a car parking lot. The server finds the ‘best’ available car parking lot for this particular user, based on his/her preferences specified in the user profile, and (optionally) reserves it. Driving directions are then sent to the user along with a detailed map, e.g., by utilizing the Google Map app with an Android API.

**Figure 8.**
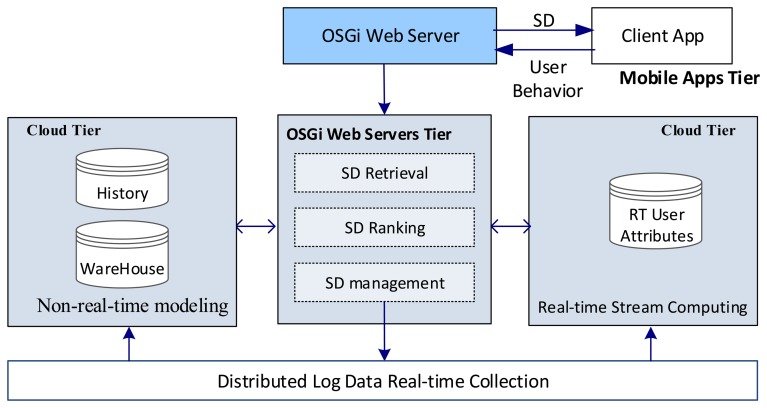
The main components of the cloud-based car parking system.

Figure 8 depicts the system's main components. The cloud tier includes a real-time stream computing part for user behavior real-time updating, parking fees charging, *etc.*; and a non-real-time modeling part for data mining and warehouse management. The distributed log data collection part acts as a high-speed data pipe in the system. The OSGi web servers tier in the centre acts as a bridge between the other two tiers. Different car parking services could be provided; these are described by their service descriptions (SD) [17,29].

### 3.2. Implementation

#### 3.2.1. Cloud Tier

**Figure 9.**
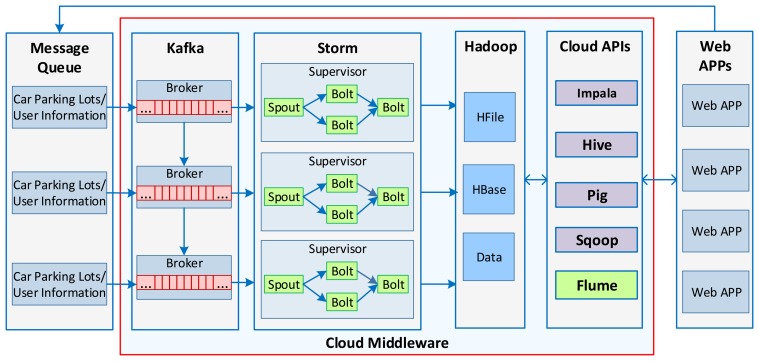
The cloud solutions for the car parking service.

Hadoop is an open-source framework for distributed processing of ‘big data’ on a number of computers using Map/Reduce programming models. It is a highly reliable and scalable for parallel processing of ‘big data’ sets. In the car parking service, some functions are offline and depend on the Map/Reduce algorithms, e.g., profile updating algorithms, *etc.*, whereas others are real-time navigation functions. The distributed dataset allows efficient data processing, whereby recommendations for the ‘best’ available car parking lots could be delivered to different users from different servers. To meet this requirement, a cloud middleware is developed with three clusters—Kafka (kafka.apache.org) [30], Storm (storm-project.net) [31], and Hadoop Distributed File System (HDFS, hadoop.apache.org) [32]—as depicted in Figure 9. Kafka is a high-throughput distributed messaging platform, used as a load-balancing cluster for parallel data loading into Hadoop. The Message Queues produce topics via multiple Brokers; topics are then consumed by the Storm Spouts. The Storm Supervisors maintain the topology, *i.e.*, when a Spout receives topics from Kafka, the Bolts start processing the data (*i.e.*, filtering, clustering, mining, *etc.*) in real time. Then the useful dataset is serialized to HBase in the Hadoop cluster. With the column-based HBase database, web applications can access the database with *put*, *scan*, *add*, *get* operations in real time. Pig, Sqoop, Hive (hive.apache.org) [23], Cloudera Impala (ccp.cloudera.com) [33], and Flume (flume.apache.org) [34] are utilized for data mining purposes.

With these cloud solutions, the information about car parking lots—along with other supplementary user information—is collected in real time, and the service is accessible via cloud APIs, *i.e.*, Hive, Pig, Impala, *etc*.

**Figure 10.**
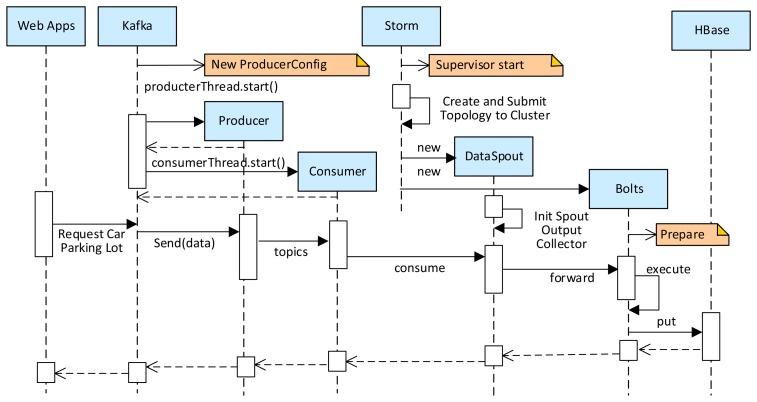
The sequence diagram of the web applications posting datasets to the cloud.

Figure 10 shows the sequence diagram of the web applications posting datasets to the cloud. In the Kafka module, firstly a *ProducerConfig* is initialized with the following parameters: A *broker* list for determining the *leader* of each *topic*, a *serializer* for encoding the message for transmission, and a *partitioner* for defining the topic of the message. Finally, a *kafka.javaapi.producer* is generated, which finds the lead broker for a particular topic and partition, fetches the corresponding messages and metadata, and sends them to Storm. This *publish-subscribe* messaging mechanism used provides scalable solution for loading data into the cloud. In the Storm module, firstly a Storm supervisor is started on all nodes of the cluster, and the designed *topology* is submitted by Storm's *backtype.storm.StormSubmitter*. Then the *DataSpout* and *Bolts* are created and initialized. When a new message arrives (e.g., from an InfoStation or a user), the *backtype.storm.task.OutputCollector* in the *DataSpout* forwards the message to *Bolts*, and after some real-time computing, the data is saved to HBase with Data Access Objects (DAO), *i.e.*, the *CarParkingDataDao*, *UserDataDao*, *etc*.

**Figure 11.**
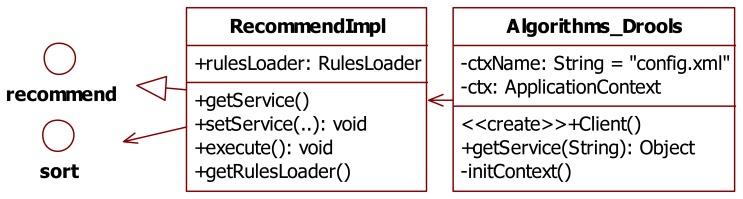
The UML diagram of drools-based recommendation classes.

Figure 11 shows the UML diagram of the Drools main recommendation classes, used in the rule engine part. The *RecommendImpl* class implements the interface *recommend* and gets the recommended car parking SDs via a sort interface. With this interface design pattern, when a new rule is applied to the system, the system does not need to be recompiled thus ensuring loose-coupling. Defined by the Drools rule language, the *rulesLoader* class is used to load the “.drl” file and to describe multiple rules, queries, and functions. The *Algorithms_Drools* class defines the application context, creates car parking user (client) data, and provides *getService* functions to the user.

#### 3.2.2. OSGi Web Servers Tier

**Figure 12.**
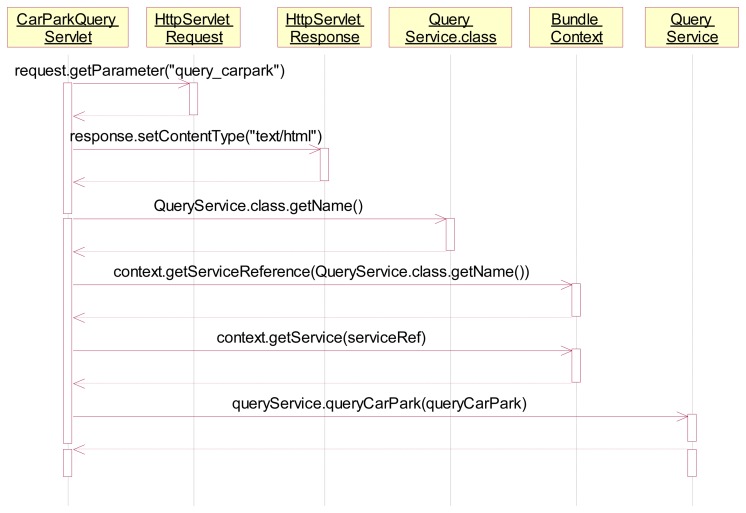
The car-parking-lot query function with OSGi bundles.

Due to the great number of OSGi-based web applications running in this tier, the web application bundles should be able to dynamically register/remove themselves to/from the container. The implementation steps of the car-parking OSGi bundles are listed below:

(a) Define the car park *QueryService* bundle's interface and add it to the *export-package* in the *MANIFEST.MF*.
(b) Implement the *QueryService* interface and return the car-parking-lot description. (The corresponding operations include the creation of a *ServiceRegistration* object in the OSGI's activator, registration of the *QueryService* in the *start* function, and removal of the *QueryService* from the *stop* function.)
(c) Implement the car park *QueryService* HTTP response bundle. The web's Servlet objects are registered as *HttpService* in the Activator, and an *org.osgi.framework.BundleContext* is linked to the Java Servlet for providing the car-parking-lot query function (Figure 12).

**Figure 13.**
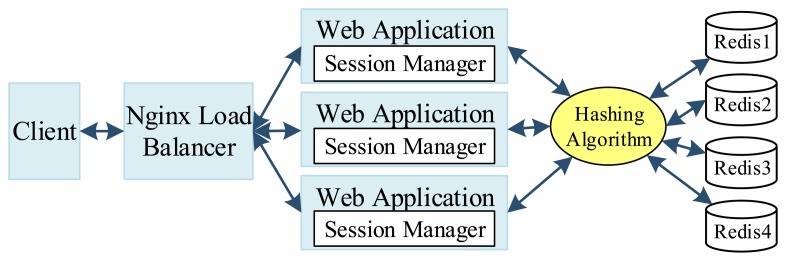
The load balancer, session manager and distributed redis in the OSGi web servers tier.

For web logs collection, the Hadoop-based Flume is used for moving large amounts of log data to the cloud. To improve the web server's performance, a number of solutions are provided, including a Nginx load balancer [35] used to accelerate the users' responses, a consistent hashing algorithm operating in the distributed Redis memory database, and session managers synchronizing the sessions of different web applications (Figure 13). The Zookeeper is used to maintain the status of the session managers to ensure data synchronization. The hashing algorithm is designed, based on the Java's *ConcurrentHashMap* function, for distribution of reading/writing operations on the Redis cluster.

#### 3.2.3. Mobile Apps Tier

**Figure 14.**
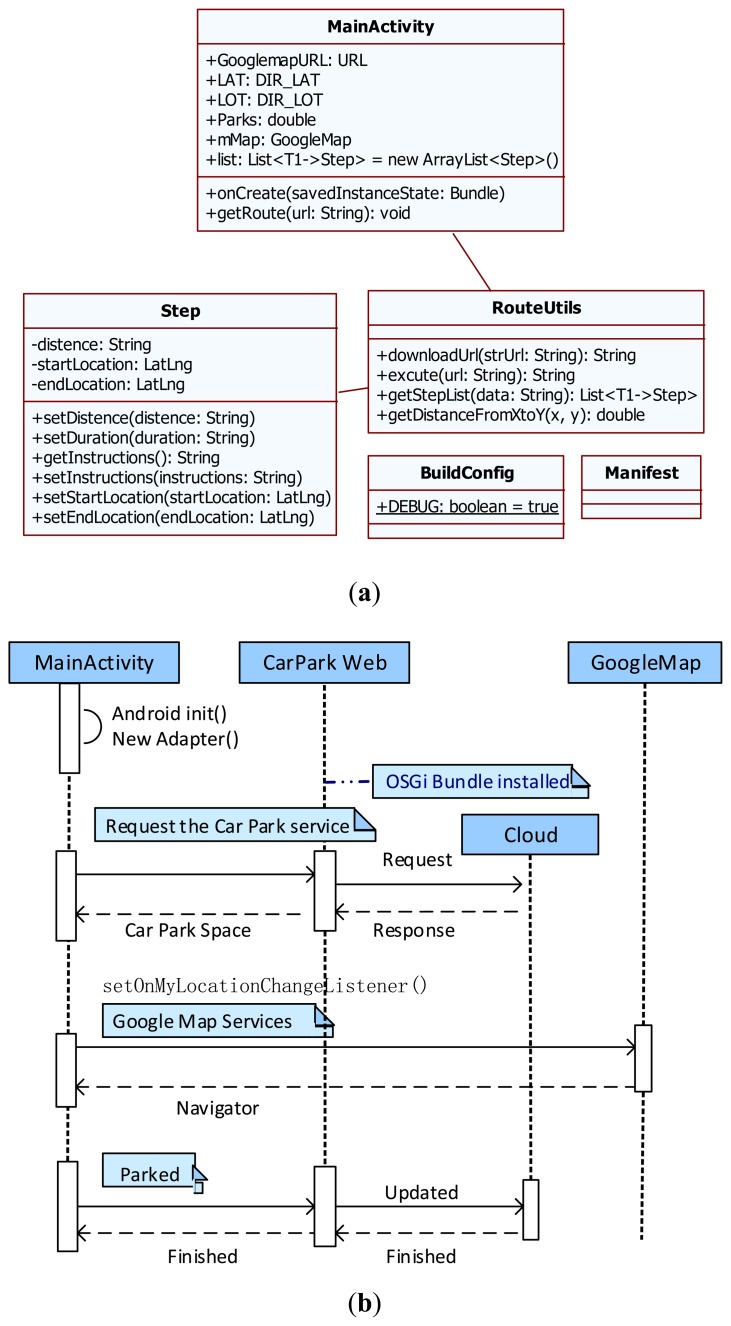
(**a**) The class diagram of the android car parking mobile App; (**b**) The sequence diagram of the car parking main activity.

The developed Android car parking mobile app is a single-view application, mainly including three Java classes: (i) an Android Activity, named *MainActivity*, which provides the user interface; (ii) a plain old Java object (POJO), named *step*, which defines the route proprieties with *get* and *set* methods; and (iii) a controller manager, named *RouteUtils*, which obtains data from the web tier. When the *RouteUtils* object is active, it communities with the Google Map APIs and sends out the car parking service request to the OSGi web server. After obtaining the car parking information from the cloud tier, a *Step* object is returned and the Google Map navigation is started. Figure 14a illustrates the class diagram of the Android car parking app. Figure 14b depicts the sequence diagram of the *MainActivity* function.

### 3.3. Results

**Figure 15.**
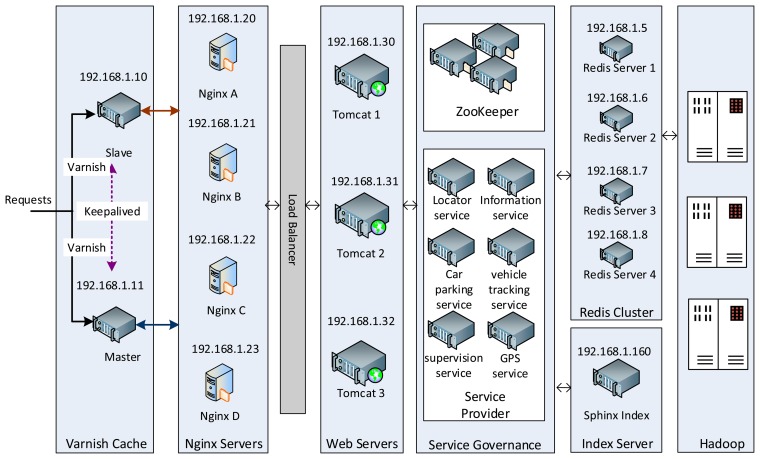
A high-level view of the car parking system deployment.

The system development follows the personal software process (PSP) methodology [36]. Test-driven and feature-driven development methods are used for the PSP. Figure 15 shows the deployment of the car parking system, which includes the following components: A Varnish cache [37], acting as a reverse proxy server for accelerating the HTTP responses; a web server, based on a number of Nginx servers for load balancing; a service consumer and producer, acting as a distributed service framework for service governance (sync-over-async and request-response messaging functions, load-balancing/failover/clustering capabilities with remote procedure call (RPC) mechanism, *etc.*); a Zookeeper [38] service register, used for car parking services registration and service events publishing/subscription; a Redis cluster, consisting of a number of Redis servers providing distributed operations for other tiers; and a Sphinx index [39], providing a full-text search functionality for users/web applications.

#### 3.3.1. Cloud Tier

**Figure 16.**
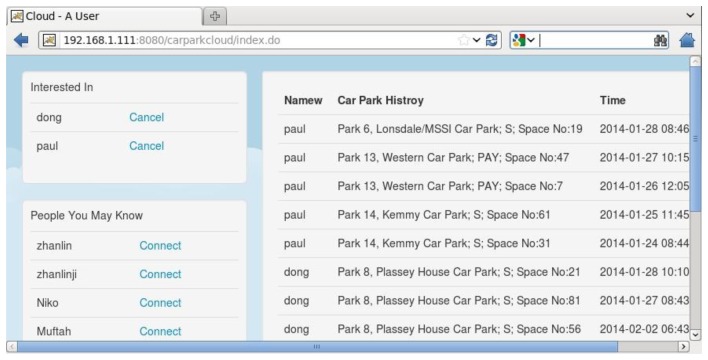
A sample car-parking usage history GUI in the cloud tier.

In the cloud tier, four servers (Master, Slave1, Slave2, and Slave3) with installed Hadoop 1.2 are used. Each server was installed on an Intel XEON PC with E3-1220L CPU and 8 GB RAM. The message size was set to 200 bytes. The Master serves as the Name node for the Hadoop, and the others are Data nodes. Three Kafka servers run on each slave node and one Storm supervisor runs on each server. The Storm *nimbus* and user interface are initialized in Slave1. The defined topology is submitted in Slave1 by a *Storm jar* command. The car parking lots and users are described by a Google's protocol buffer. For demonstration purposes, 2000 car parking lots and 2000 users are randomly generated and stored in the HBase database. The cloud-tier applications, including the car parking lots management, Map/Reduce algorithms scheduling, personal profiles maintenance, *etc.*, are hosted on the InfoCentre. For security reasons, the cloud-tier applications can't be accessed by the end users; they can only be accessed by the car-parking service administrator or particular applications in the web servers tier. Figure 16 shows a fragment of the car parking usage history.

**Figure 17.**
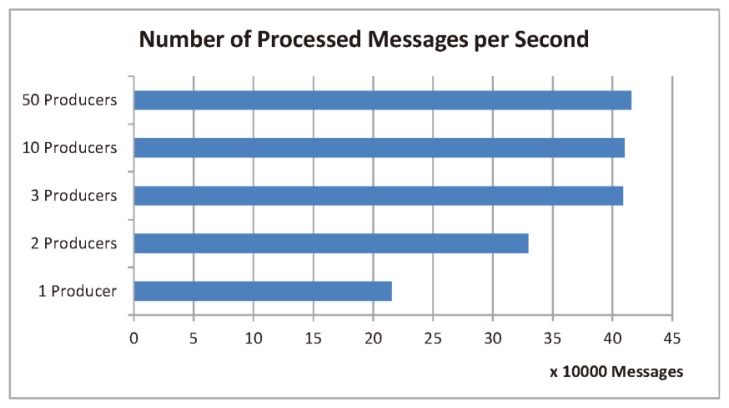
The number of processed messages per second for different number of kafka producers (clients) in the cloud tier.

Five tests were conducted with different number of Kafka producers (clients). Each producer sends a number of messages to the cloud in an asynchronous manner. The *snappy* was utilized as a compression algorithm. The batch size was set to 2000 messages and the Kafka partitions number to 9. The system throughput, *i.e.*, the average number of processed messages per second, achieved in each test is depicted in Figure 17. The results show that the optimal number of producers is 3, as going beyond this number does not result in a significant increase in the system throughput. For this optimal case, the achieved throughput is about 400,000 messages per second, which is sufficient for an all-Ireland national car parking system.

#### 3.3.2. OSGi Web Servers Tier

**Figure 18.**
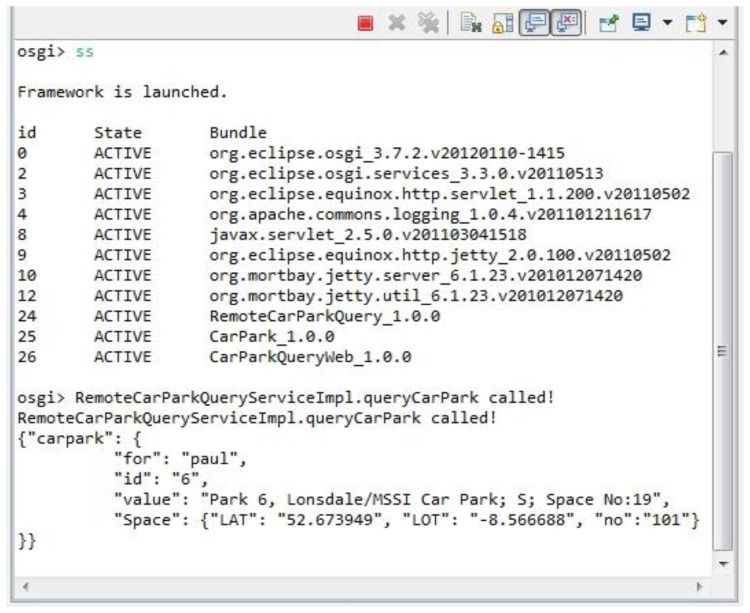
The car parking query service's OSGi bundle output.

In this tier, three web servers (*cf.* Figure 15) are set up to run with CentOS 6.5 and Equinox OSGi container [40]. One of the servers is configured with a Nginx load balancer. Bundles are deployed on these three web servers. Figure 18 shows the *RemoteCarParkQuery*, *CarPark*, and *CarParkQueryWeb* bundles in the OSGi environment. The dependent bundles, *i.e.*, OSGi service, Servlet, Logging, Jetty, *etc.*, are also active in the environment. When a *queryCarPark* request is received, a bundle forwards the request to the cloud tier. When the response arrives, it is formatted with JavaScript Object Notation (JSON) and is sent back to the Android client application.

**Figure 19.**
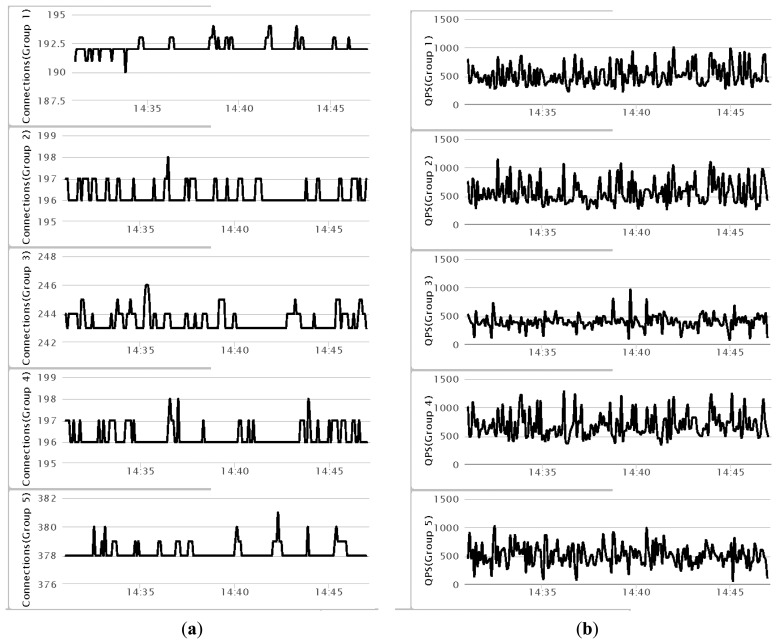
The performance visualization of the distributed Redis framework: (**a**) Number of connections; (**b**) queries per second, QPS.

The *Redis* cluster is deployed on four computers with IP addresses 192.168.1.5∼8, (*cf.* Figure 15). Five Redis servers run on each computer on ports 5001∼5005, respectively. Each of these ports is dedicated to one particular type of car parking service, *i.e.*, locator service, information service, GPS service, vehicle tracking service, and supervision service. When a request is delivered to the Redis cluster, it will be dispatched to a Redis server by means of a consistent hashing algorithm. To demonstrate the distributed properties, at the OSGi web servers tier, about 1200 connections are utilized to send out *mobile?java&{service}* reading requests from the web applications to the system. From these, 16% are for service 1, 16.5% for service 2, 20% for service 3, 16.5% for service 4, and 31% for service 5 (Figure 19a). The total number of queries per second (QPS), initiated to the system, is around 2700, whereby 29% go to computer 1, 30% to computer 2, 21% to computer 3, and 20% to computer 4. Figure 19b shows the QPS for each group of services.

#### 3.3.3. Mobile Apps Tier

**Figure 20.**
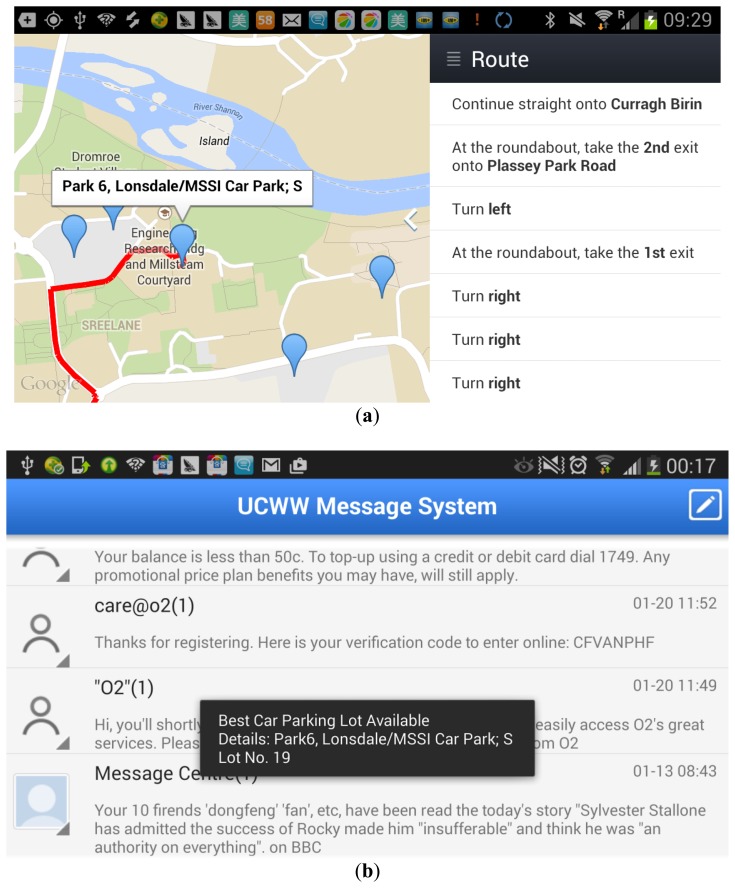
The Car Parking Android Mobile App for navigation: (**a**) for GPS-enabled mobile devices; (**b**) for non-GPS-enabled mobile devices.

In this tier, when a user in a car enters the university campus through one of its gates, the car parking mobile app, installed on the user mobile device, will send an automatic HTTP request through the gate's wireless access point toward a web server, and a JSON response will be returned, containing information about the ‘best’ available car parking lot. For a GPS-enabled mobile device, a *RouteUtils* generates travel directions (steps) to be followed by the driver, and displays them on the Google Map as shown in Figure 20a. For a non-GPS-enabled mobile device, a Short Message Service (SMS) application displays the car parking lot information to the user, as shown in Figure 20b.

**Figure 21.**
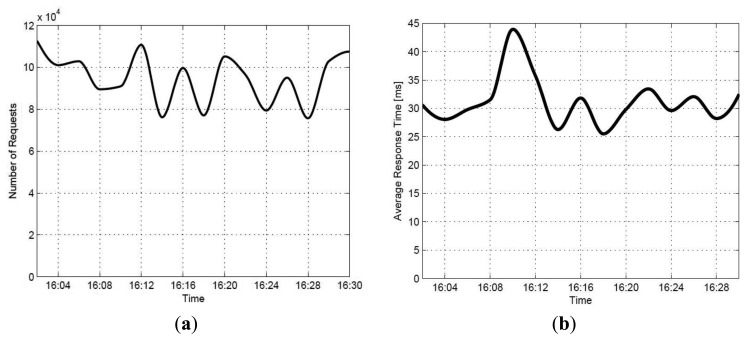
(**a**) The number of requests as a function of time; (**b**) The system's average response time.

To evaluate the mobile app's performance, clients are made to submit 1.5 million requests (in total) to *mobile?java&{carparking}&{JSON}* for a period of 1800 s (*i.e.*, the average number of concurrent requests per second is 833). The total network throughput is 7200 MB (from 16:00 to 16:30). Figure 21 illustrates the number of requests as a function of time and the system's average response time.

The results confirm the ability of the developed car parking system to serve a great number of clients, by keeping the average response time at the millisecond level. The minor fluctuations in the system's response time are mainly due to the packet delays caused by the network.

## 4. Conclusions

An IoT cloud-based intelligent car parking system has been described in this paper. Considered as an important component of an Intelligent Transport System (ITS) for smart cities, the car parking system is built with three layers: Sensor-, communication-, and application layer. The system middleware and corresponding operational platform have been described. In the implementation part, a sample car parking service for a University campus has been considered along with the supporting cloud applications, OSGi-based web applications, and Android mobile applications. The service provides the user (the driver) with information about the ‘best’ available car parking lot, which is communicated back to the user's mobile app, following the Always Best Connected and best Served (ABC&S) paradigm.
